# The Prevalence of Chromosomal Deletions Relating to Developmental Delay and/or Intellectual Disability in Human Euploid Blastocysts

**DOI:** 10.1371/journal.pone.0085207

**Published:** 2014-01-07

**Authors:** Wenyin He, Xiaofang Sun, Lian Liu, Man Li, Hua Jin, Wei-Hua Wang

**Affiliations:** 1 Key Laboratory of Major Obstetrics Diseases of Guangdong Province, The Third Affiliated Hospital of Guangzhou Medical University, Guangdong, China; 2 Pacgenomics Inc., Village Medical Center, Thousand Oaks, California, United States of America; 3 New Houston Health, Houston, Texas, United States of America; 4 Vivere Health, Franklin, Tennessee, United States of America; Institute of Zoology, Chinese Academy of Sciences, China

## Abstract

Chromosomal anomalies in human embryos produced by in vitro fertilization are very common, which include numerical (aneuploidy) and structural (deletion, duplication or others) anomalies. Our previous study indicated that chromosomal deletion(s) is the most common structural anomaly accounting for approximately 8% of euploid blastocysts. It is still unknown if these deletions in human euploid blastocysts have clinical significance. In this study, we analyzed 15 previously diagnosed euploid blastocysts that had chromosomal deletion(s) using Agilent oligonucleotide DNA microarray platform and localized the gene location in each deletion. Then, we used OMIM gene map and phenotype database to investigate if these deletions are related with some important genes that cause genetic diseases, especially developmental delay or intellectual disability. As results, we found that the detectable chromosomal deletion size with Agilent microarray is above 2.38 Mb, while the deletions observed in human blastocysts are between 11.6 to 103 Mb. With OMIM gene map and phenotype database information, we found that deletions can result in loss of 81-464 genes. Out of these genes, 34–149 genes are related with known genetic problems. Furthermore, we found that 5 out of 15 samples lost genes in the deleted region, which were related to developmental delay and/or intellectual disability. In conclusion, our data indicates that all human euploid blastocysts with chromosomal deletion(s) are abnormal and transfer of these embryos may cause birth defects and/or developmental and intellectual disabilities. Therefore, the embryos with chromosomal deletion revealed by DNA microarray should not be transferred to the patients, or further gene map and/or phenotype seeking is necessary before making a final decision.

## Introduction

Chromosome anomalies occur when there is an error during meiosis or mitosis [1]. High proportions of human embryos resulting from in vitro fertilization (IVF) have abnormal chromosomes, mainly raised from meiosis [2]–[5]. Recent use of all-chromosome DNA microarray indicates that most of these abnormalities are numerical anomalies, i.e. aneuploidy [6]–[9]. However, many embryos (either aneuploid or euploid) had structural anomalies, which include chromosomal deletion and/or duplication [9]. When aneuploidy, with or without additional structural anomalies, was detected in human embryos, regardless of which chromosome, these embryos are considered abnormal and are not transferred to patients. However, when a euploid embryo has structural anomalies, especially in case of microdeletion(s), which are generally considered to be more pathogenic than microduplication(s) [10], [11], it is difficult for clinical physicians and patients to make a decision whether the embryo is transferrable or not.

There are many kinds of deletions in human chromosomes: some are related with diseases, while others may not [12]–[16]. For examples, patients with 1q21.1 deletion syndrome have delayed development, intellectual disability, physical abnormalities, and neurological and psychiatric problems [17]. These symptoms may be related to the loss of genes in this region, such as *ACP6, BCL9, CHD1L, FMO5, GJA5, GJA8, GPR89B and HYDIN* [omim.org]. However, the exact gene in some deletion syndromes has not been identified. For example, 1p36 deletion syndrome is caused by a deletion of genetic material from a specific region in the short arm of chromosome 1 [18]. The symptoms of this disorder include intellectual disability, distinctive facial features, and structural abnormalities in several body systems [18]. However, the gene(s) that is related with these syndromes has not been identified. It is assumed that the pathogenesis is caused by the dose effect of the genetic material loss.

Infertility has become one of the major health problems in humans, especially in the recent decades [19]. It has been estimated that up to 4% of human newborns are resulting from assisted reproductive technology (ART) [20]. Although some studies indicated that there is no obvious health problem and developmental problem in the children derived from ART as compared with natural conception [21]–[23], ART may provide a better platform for screening the embryos, and this cannot be done during a natural conception, in which prenatal diagnosis may be necessary. In the future, with the development of DNA sequencing and gene mapping, it will be possible to find all genetic problems in the samples biopsied from preimplantation embryos, thus ART may become a technology not only for infertility treatment, but also for prevention of the occurrence of genetic diseases in a quiet early stage in advance of the pregnancy. This has already been performed in some patients with known inherited diseases, such as α- and β- thalassemia, Duchenne muscular dystrophy and spinal muscular atrophy [24]–[26].

The introduction of molecular techniques in conjunction with classical cytogenetic methods has, in recent years, greatly improved the diagnostic potential for chromosomal abnormalities [27]. In particular, array based comparative genomic hybridization (aCGH) promises a sensitive strategy for the detection of DNA copy-number changes on a genome-wide scale [28]–[33]. The resolution of detection could be as high as one million “bands” and the size of chromosomal deletion detected could be as small as 5 kb in length, such as 1 million isothermal probe NimbleGen platform [29]–[31].

Currently, there are three popular DNA microarray platforms have been used in human preimplantation genetic screening (PGS): bacteria artificial chromosomes (BAC) [7], [34], [35], single nucleotide polymorphisms (SNP) [6], [35], [36] and oligonucleotide [8], [9] DNA microarray platforms. However, these array platforms do not show the correlation between the chromosomal deletion and gene loss or related corresponding phenotype(s) directly. This raises the question if the embryos with some chromosomal deletion(s) are transferrable or not. If there are important gene losses in the deleted region, these embryos should not be transferred to the patients. However, if there is not an important gene in the deleted region, the embryos may be “normal” for transfer.

Application of whole genome copy number variants (CNVs) analysis indicated that individuals with developmental disability, such as developmental delay and intellectual disability, accounts for up to 14% of population [37]–[39]. The data is mainly obtained from the investigation on children, and this technology recently has been applied to prenatal diagnosis [40], but the clinical data is still missing. Furthermore, because the analysis is applied to the samples collected from either children or fetuses, it cannot avoid the occurrence. By contrast, if a detailed CNVs analysis is performed on the preimplantation embryos, it would be possible to prevent the occurrence in this population. Because high proportions of human embryos produced by IVF are aneuploidy, it is possible that CNVs, such as chromosomal deletions, are also common in these embryos.

In order to answer these questions, we found that the oligonucleotide microarray platform developed by Agilent can provide such information, which is very useful for detailed analysis of chromosomes and genes, as well as phenotypes in the deleted regions. The data revealed by the Agilent array platform could be also linked to the OMIM gene map and phenotype database, which contains the most updated gene map information of human chromosomes. From this database, it is possible to look into the exact location and size of each deletion, and scientists can also find the genes in the deleted region in each chromosome and their phenotypes. Although the Agilent microarray platform has been used for prenatal diagnosis in human clinics [40], it has not been applied to human PGS. Therefore, in this study, we first validated the Agilent DNA microarray platform using pre-known embryo biopsy samples tested by either BAC platform or oligo platform. Then we re-tested some euploid samples that had been previously screened to have chromosomal deletion(s), and we further linked these chromosomal deletions to the OMIM gene map and phenotype database to investigate if these deletions are clinically significant, paying particular attention to the known clinical significance of the CNVs related to developmental delay and/or intellectual disability.

## Materials and Methods

### Ethics Statement

All DNA samples used in this study were from previous PGS. Patients undergoing previous IVF and PGS signed written consents for embryo biopsy and aneuploidy screening. The study was approved by institute research committee and medical ethics committee of the Third Affiliated Hospital of Guangzhou Medical University, which has the same functions as other Institutional Review Boards. When the committee reviewed the research project, they understood that the study involved the review of existing data, documents, records, and diagnostic specimens and re-test and analysis of the samples in such a manner that the subjects could not be identified directly or through identifiers linked to the subjects.

### Searching of Chromosomal Deletion and Genetic Diseases

For seeking chromosomal deletions and their relationship with genetic diseases, we mainly used a website from NIH, USA (www.ghr.nl.nih.gov). Chromosomal deletions in all 23 pairs of chromosomes were searched for, including name of disease (syndrome), location of chromosomal deletion, occurrence, main problems (phenotypes) and main genes in the deleted region that associated with the deletion.

### Validation of Agilent Microarray by Comparing with Oligo Nimblegen and Bac Bluegnome Platforms

Previous embryo DNA samples tested by either NimbleGen oligo or BlueGnome BAC microarrays were re-analyzed with Agilent microarray platform. The methods for NimbleGen and BlueGnome array platforms were described in our previous studies [8], [9]. The Agilent array was performed based on the procedures described below. Amplified samples were labeled with Cy3 using SureTag DNA labeling kit (Agilent). Labeled samples were then mixed with Cy5 control labeled samples. The labeled samples and controls were purified with SureTag DNA labeling purification column (Agilent), dried, dissolved in hybridization buffer containing Cot-1 DNA, 10×aCGH blocking agent, and 2×HI-RPM Hybridization buffer (Agilent), and loaded onto SurePrint G3 human CGH 8×60K Oligo Microarray (Agilent). After overnight hybridization at 65°C, microarrays were washed following Agilent washing protocol. Microarrays were scanned with SureScan Microarray Scanner (Agilent) at 3 µM. Scanned images were analyzed by Cytogenomics software (Agilent), and the normalized ratio of each sample versus the control was retrieved following Agilent CGH data analysis protocol. The result of each sample's whole genome view is presented. After Agilent array, the results on each sample were compared with the results obtained by NimbleGen arrays or BlueGnome arrays.

### Agilent Microarray Analysis of Previous Known Deletions in Coriell Cells and Blastocyst Biopsies

Coriell cell lines were obtained from Coriell Institute (New Jersey, USA) and human embryo (blastocyst) biopsy samples were obtained from PacGenomics Inc., which performs human PGS in USA. These samples were re-tested with Agilent microarray platform using the methods described above.

### Gene Map and Phenotype Seeking

For gene map and phenotype information, we used OMIM website (www.omim.org) and Agilent software that is directly linked to Agilent database when the array data was obtained. The genes in the deleted chromosomal segment were automatically displayed and associated phenotypes (if they are already known) related to the specific genes is also displayed in the table. From the data in the table, we investigated possible genetic problems. After searching for the database, we made the decision of whether the euploid embryos with chromosomal deletion were normal or abnormal.

## Results

### Chromosomal Deletion and Human Diseases

As shown in Table 1, we found that 28 chromosomal deletions or microdeletions have been identified and recorded to be related with human genetic diseases. All deletions have been found in somatic cells after live birth. Some of these deletions were very rare, such as 1q21.1 deletion syndrome, 2q37 deletion syndrome, Langer-Giedion syndrome and 15q24 microdeletion, while some occur in high proportions, such as Y chromosome deletion (azoospermia factor in male population), which can be as high as 1∶2,000∼1∶3,000. Another example of high occurrence is 22q11.2 deletion syndrome, which occurs 1∶4,000 in population and can cause problems in many parts of the human body.

**Table 1:** Chromosomal deletions and diseases.

Name of diseases	Location	Occurrence	Main problems	Main Genes
1p36 deletion syndrome	1p36.13-33	1∶5,000-10,000	Intellectual disability	unsure
1q21.1 deletion syndrome	1q21.1	rare	Delayed development, Intellectual disability	*ACP6, BCL9, CHD1L, FMO5, GJA5, GJA8, GPR89B, HYDIN*
2p16.1-p15 deletion syndrome	2p16.1-p15	rare	Intellectual disability, metal retardance, skull and facial anomalies	*DEL2P16, I-P15*
2q37 deletion syndrome	2q37	rare	Hypotonia, Delayed development, Intellectual disability	unsure
Wolf–Hirschhorn syndrome	4p16.3	1∶50,000	Facial appearance, Delayed development, Intellectual disability	*WHSC1*, *LETM1*, *MSX1*
5p deletion syndrome	5p15.3, 15.2	1∶20,000-50,000	Delayed development, Intellectual disability, small head	*CTNND2*
Williams syndrome	7q11.23	1∶7,500-20,000	Development Disorder, Intellectual disability, learning problem	*CLIP2*, *ELN*, *GTF2I*, *GTF2IRD1*, *LIMK1*
Langer-Giedion syndrome	8q24.1	rare	Bone abnormal	*EXT1*, *TRPS1*
9q22.3 microdeletion	9q22.3	rare	Delayed development, Intellectual disability, some physical abnormal	*PTCH1*
Kleefstra syndrome	9q34.3	Unknown, rare	Delayed development, Intellectual disability	*EHMT1*
Potocki-Shaffer syndrome	11p11.2	rare	Development of bones, brain and other organs	*EXT2*, *ALX4*
Jacobsen syndrome, 11q terminal deletion disorder	11q24.1	1∶100,000	Delayed development	unsure
WAGR syndrome	11p13	1∶500,000	Development of many body	*BDNF*, *PAX6*, *WT1*
Retinoblastoma	13q14	rare	Intellectual disability, slow growth, and characteristic facial features	*RB1*
15q13.3 microdeletion	15q13.3	1∶40,000	Intellectual disability, epilepsy, schizophrenia, or autism spectrum disorders.	unsure
15q24 microdeletion	15q24	Very rare	Intellectual disability and delayed speech development	unsure
Sensorineural deafness and male infertility	15q	unknown	Hearing loss and an inability to father children.	*CATSPER2*, *STRC*
Prader–Willi syndrome	15q11-13	1∶10,000-30,000	Many parts of the body	*OCA2*
16p11.2 deletion syndrome	16p11.2	3∶10,000	Developmental delay and intellectual disability	unsure
Rubinstein-Taybi syndrome	16p13.3	1∶100,000-125,000	Short stature, moderate to severe intellectual disability, distinctive facial features, and broad thumbs and first toes	*CREBBP*
Miller-Dieker syndrome	17p	rare	Abnormal brain development	*PAFAH1B1*, *YWHAE*
Koolen-de Vries syndrome	17q21.31	1∶16,000	Developmental delay and mild to moderate intellectual disability	*KANSL1*
Smith-Magenis syndrome	17p11.2	1∶15,000	Intellectual disability, delayed speech and language skills, distinctive facial features, sleep disturbances, and behavioral problems	*RAI1*
Alagille syndrome	20p12	1∶70,000	Liver, heart, and other parts of the body	*JAG1*, *NOTCH2*
22q11.2 deletion syndrome	22q11.2	1∶4,000	Many parts of the body	*T*, *TBX1*
Phelan-McDermid Syndrome)	22q13.3	few	Many parts of the body	*SHANK3*
Microphthalmia with linear skin defects syndrome	Xp22	rare	Mainly affects females, microphthalmia	*HCCS*
Azoospermia factor/Y chromosome infertility	Yq11.23	1∶2,000-3,000 male	Azoospermia, oligospermia	*USP9Y*

When we examined the main problems in these chromosomal deletions, as shown in Table 1, we found that 17 out of 28 chromosomal deletions were related with intellectual disability and/or delayed development. We also found that genes have been identified in 22 of the deletions and have not been identified only in 6 deletion syndromes. In some deletion syndromes, only one gene is involved, such as 5p deletion syndrome and 9q23.2 microdeletion, while in some deletion syndromes, a few genes are involved, such as 1q21.1 deletion syndrome, William syndrome, Wolf-Hirschhorn syndrome and WAGR syndrome.

### Application of Agilent Array Platform in Human PGS

**Figure 1:**
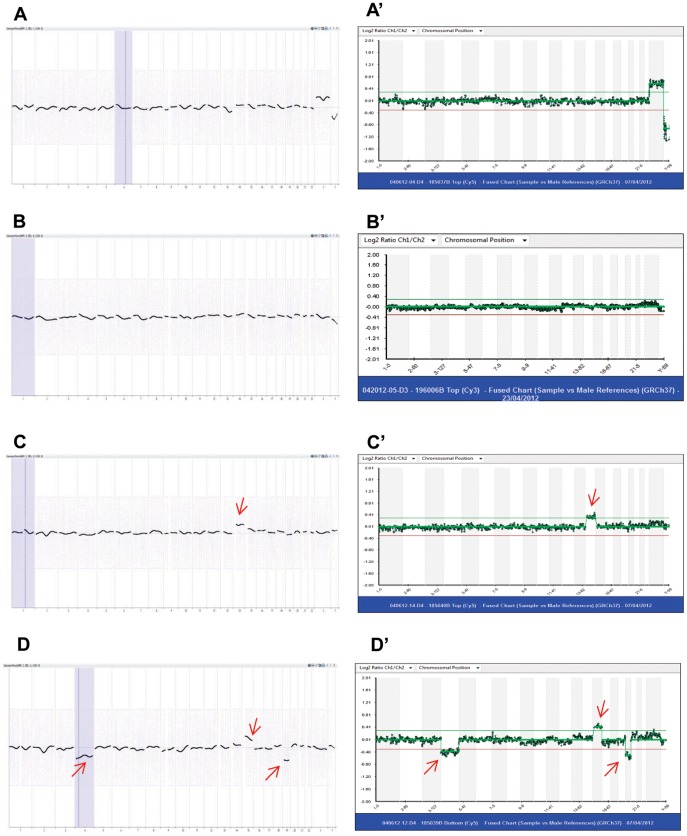
PGS charts of human blastocyst biopsies from Agilent oligo and Illumina BAC DNA array platforms. Charts in left column are from Agilent and charts in right column are from Illumina BAC platforms. **A** and **A**': 46 XX; **B** and **B**': 46 XY; **C** and **C**': 47 XY, +14; **D** and **D**': 45 XY, −4, +15, -19. Arrows indicate chromosomal errors. Data matched between two array platforms.

**Figure 2:**
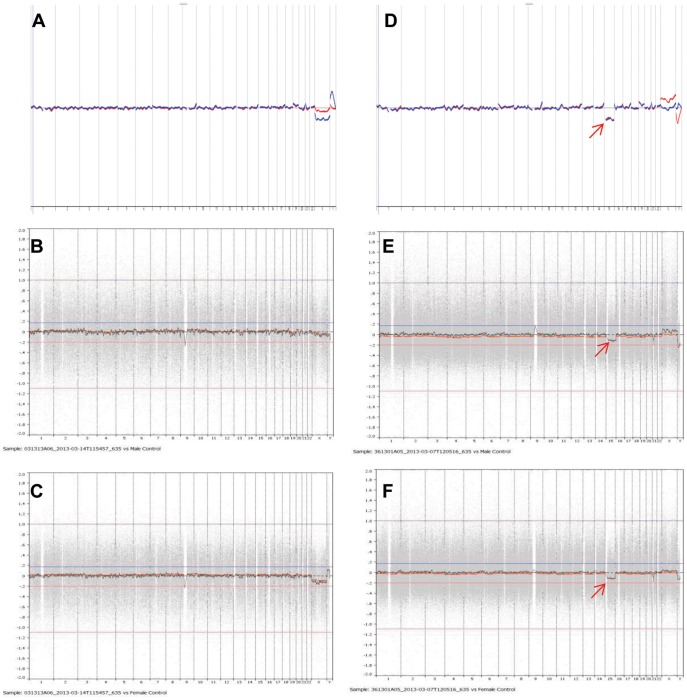
PGS charts of human blastocyst biopsies from Agilent and NimbleGen oligo DNA array platforms. Charts in **A** and **D** are from Agilent and charts in **B**, **C**, **E** and **F** are from NimbleGen platforms. **A**, **B** and **C**: 46 XY; **D, E** and **F**: 45 XX, -15. Arrow indicates chromosomal error. Data matched between two array platforms.

For validation of Agilent microarray platform in human PGS, we used 28 previous PGS samples (16 with BAC platform and 12 with NimbleGen array platform) including 3 euploid samples and 25 aneuploid samples. As shown in Table 2 and Figures 1 and 2, all samples had matching results between different array platforms.

**Table 2:** Validation of chromosomes with three array platforms in human embryo samples.

Sample	Agilent platform	BluGnome platform	NimbleGen platform
1	46, XX	Match	NA
2	46, XY	Match	NA
3	47, XXY	Match	NA
4	47, XY, +14	Match	NA
5	45, XY, −4, +15, −19	Match	NA
6	45, XX, −2	Match	NA
7	45, XX, −21	Match	NA
8	47, XY, +22	Match	NA
9	46, XY, +7, −16	Match	NA
10	51, XX, +11, +13, +14, +16, +18	Match	NA
11	48, XX, +7, +11, −12, −14, +15, +17	Match	NA
12	43, XXY, −4, +5, −6, −8, −9, −13, −18, +19, −21, +22	Match	NA
13	47, XX, +6, +12, +17	Match	NA
14	45, XY, −19	Match	NA
15	47, XX, +6, del 20q	Match	NA
16	46, XY, −10, +16	Match	NA
17	45, XO	NA	Match
18	46, XY	NA	Match
19	45, XX, −22	NA	Match
20	42, XX, −2, −4, −13, −22	NA	Match
21	45, XY, −22	NA	Match
22	47, XY, +16	NA	Match
23	45, XX, −3, −8, +16	NA	Match
24	45, XY, −19	NA	Match
25	45, XX, −15	NA	Match
26	46, XX, del Yp, dup Yq11	NA	Match
27	45, XO, −16, +21	NA	Match
28	44, XY, −15. −22	NA	Match

### Analysis of Chromosomal Deletion in Human Coriell Cells and Blastocyst Biopsies

As shown in Table 3, when 5 Coriell cell lines with known chromosomal deletion (13.2, 6.7, 6.16, 2.38 and 1.18 Mb) were examined with the Agilent array platform, we found that the smallest size that Agilent array can detect is 2.38 Mb. A sample (11p11.2) with a deletion size of 1.18 Mb was not detected with this array platform. When 15 chromosomal deletion samples from human blastocyst biopsies were re-examined (previously were examined with NimbleGen microarray platform) with Agilent array platform, all chromosomal deletions were detected and had matching results between NimbleGen and Agilent platforms. From these 15 human embryo samples, we found that chromosomal deletions were between 11.6–103 Mb, which were larger than the smallest detectable size (2.38 Mb) by Agilent array platform.

**Table 3:** Detailed analysis of microdeletions in the examined samples with Agilent DNA microarray.

Sample number*	Deletion location	Size (Mb)	Detected or not	Gene location	# of genes with phenotype	Total # of genes in the region	Example of known syndrome	Final decision of embryo
**1**	8p21.1	13.2	Yes	28,757,631–42,038,519				NA
	18q22.3	6.70	Yes	70,779,596–77,483,283				NA
**3**	18q22.3	6.16	Yes	71,772,000–77,938,705				NA
**4**	18q21.3	2.38	Yes	45,614,164–47,997,488				NA
**5**	11p11.2	1.18	No	43,726,534–44,909,506				NA
**6**	1p	71.8	Yes	2,715,805–74,579,387	149	464	1p36 deletion syndrome	Abnormal
**7**	6q	103	Yes	144,424,389–247,931,799	40	90		Abnormal
**8**	1q	64.2	Yes	100,738,994–164,995,408	94	428	1q21.1 deletion syndrome	Abnormal
**9**	7q	66.2	Yes	87,681,606–153,960,615	89	343		Abnormal
**10**	5q	78.0	Yes	100,072,432–178,168,173	90	372		Abnormal
**11**	13q	56.6	Yes	58,205,958–114,797,160	52	190		Abnormal
**12**	1q	50.8	Yes	185,451,112–236,274,449	70	244	1q21.1 deletion syndrome	Abnormal
**13**	8q	54.5	Yes	91,110,826–145,648,581	58	183		Abnormal
**14**	10p	39.0	Yes	0–39,021,455	34	114		Abnormal
**15**	4q	33.5	Yes	153,835,653–187,422,413	39	81		Abnormal
**16**	4q,	46.4	Yes	141,188,787–187,629,739	34	113		Abnormal
	5q	42.7	Yes	132,252,587–175,028,646	51	257		Abnormal
**17**	2p	64.4	Yes	0–64,497,460	67	217	2p16.1-p15 deletion syndrome	Abnormal
**18**	10p	37.6	Yes	0–37,658,898	35	112		Abnormal
	17p	11.6	Yes	4,579,097–16,212,218	39	135		Abnormal
**19**	15q	49.5	Yes	50,585,271–100,170,631	71	256	15q24 microdeletion syndrome	Abnormal
**20**	5q	87.4	Yes	93,197,999–180,652,442	90	378		Abnormal
	12q	47.2	Yes	85,432,055–132,626,501	61	201		Abnormal

1–5: Euploid Coriell cells; 6–20: Biopsied samples from euploid human embryos.

### Gene Map and Phenotype Information

**Figure 3:**
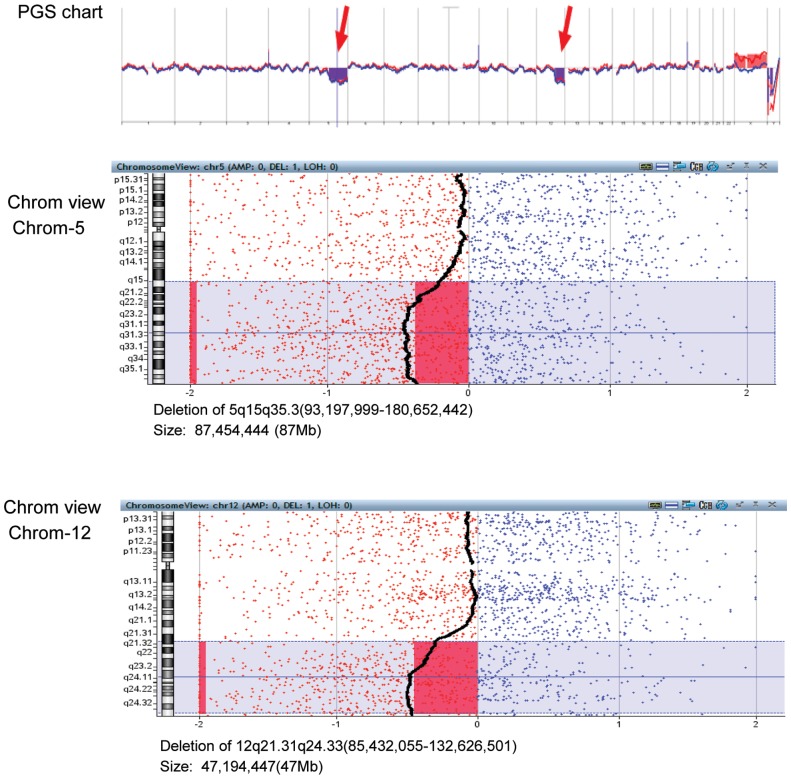
PGS chart and chromosomal view of a human blastocyst biopsy analyzed with Agilent array platform. The PGS chart shows two chromosomal deletions (arrow) in chromosomes 5 and 12. The middle and bottom charts show the chromosomal views in both chromosomes, with deletion locations and sizes.

**Figure 4:**
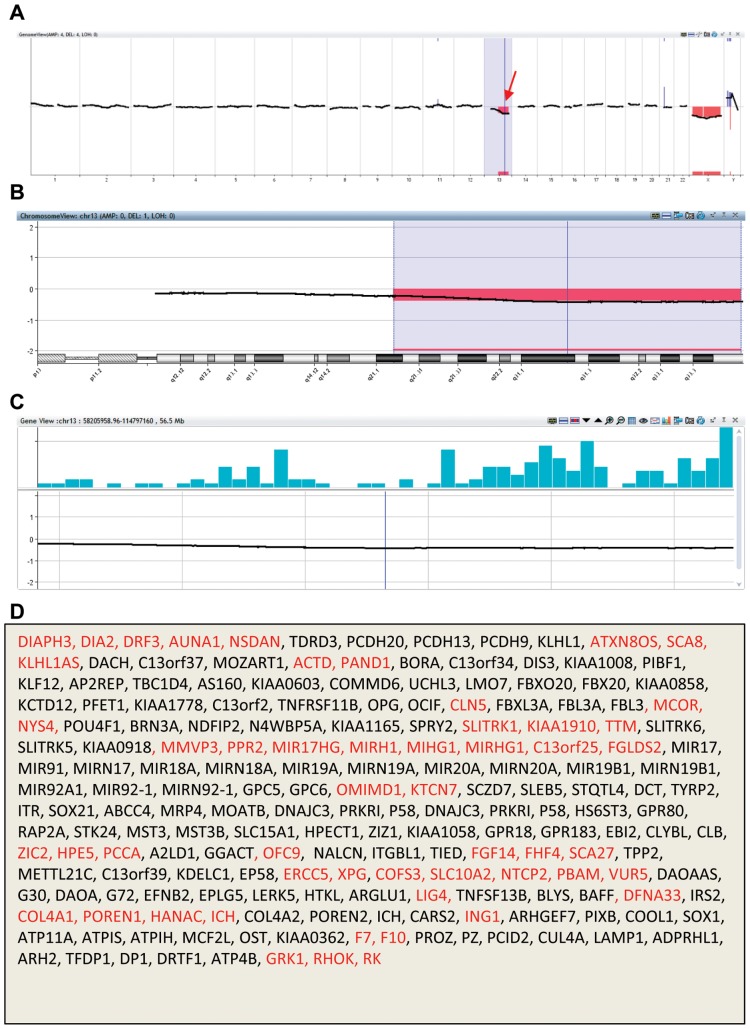
PGS chart, chromosomal view, gene view and gene list of a human blastocyst biopsy analyzed with Agilent array platform. The PGS chart (**A**) shows one chromosomal deletion (arrow) in chromosome 13. Chromosomal view chart (**B**) shows the deletion location (13q21–13q33). Gene view chart (**C**) shows the location of genes (58,205,958–114,797,160) and **D** shows the list of lost genes in the deleted region. Genes marked with red are those related with phenotypes.

When we accessed the OMIM website to search for gene map and phenotype for the deleted region in each embryo sample, as shown in Table 3, Figures 3 and 4, all genes in each deleted region and phenotypes of some genes were displayed. Some genes may have also been located in the deleted region, but associated phenotypes have not been identified. After searching for all data from 15 samples, we found that 81–464 genes lost in the deleted regions, while 34–149 of these genes were related with phenotypes. We found that 5 out of 15 samples were among the known deletion syndromes shown in Table 1, including two 1q21.1 deletion, one 1p36 deletion, one 2p16.1–p15 deletion and one 15q24 microdeletion syndromes. These five samples had CNVs related to developmental delay and/or intellectual disability (Table 1). We found that all samples had clinical significance, indicating that these embryos are abnormal and should not be transferred to the patients.

## Discussion

Aneuploidy in preimplantation human embryos is very common and is one of the major factors causing failed embryo implantation and birth defects [4], [5]. In order to increase embryo implantation and reduce birth defects, all chromosome aCGH has recently been used to screen human embryos before transfer [41]–[45] and transfer of screened embryos has significantly increased embryo implantation [6]–[9]. Chromosomal abnormalities can be either numerical (aneuploidy), or structural. Based on our previous [9] and other group's studies [6], [7], [41]–[45], it was estimated that ∼50% of human embryos produced by IVF were aneuploid and such rates increase as maternal age increases. We also found that ∼8% of human euploid blastocysts had chromosomal deletion, which is the most common chromosomal structural anomaly [9]. The chromosomal deletion in human embryos has not been paid attention to during previous embryo screening. It has been estimated that the proportion of chromosomal deletion syndromes in the human population is very high, including delayed development, intellectual disability and other severe birth defects [37]–[39].

Differing from numerical anomalies, minor structural anomalies may not cause significant syndromes after live birth. However, most of the deletion syndromes are related to delayed development and intellectual disability (Table 1). With the development of the gene map and DNA array or sequence technology, it would be possible to investigate the detailed relationship between the genes in the deleted region and genetic diseases [46]–[48]. The present study aimed to achieve this purpose in human embryos, and we found that some human embryos had the known chromosomal deletion syndromes even though the embryos were euploid, which could be detected with either NimbleGen or Agilent oligo array platforms. The benefit of Agilent array platform, besides its simple and less time-consuming operation, is its direct link to the gene map and phenotype data base. Our results, for the first time, indicate that Agilent array platform can be successfully used in human PGS, and it can detect not only aneuploidy, but also structural chromosomal anomalies, such as deletion. It also provides a platform for further investigation of the genes and phenotypes in the deleted region, allowing the physicians and clinical scientists to directly make the decision of whether these embryos are normal or abnormal. Our results indicate that, with a limited number of samples, all chromosomal deletion samples are abnormal and that these embryos should not be transferred to the patients.

With either NimbleGen or Agilent oligo microarray platform, the chromosomal deletions can be detected in human embryo biospsies. Based on our previous [9] and current study, the smallest detected size of deletion is 1.3 Mb (NimbleGen) and 2.38 Mb (Agilent). However, from the data of 15 embryo samples, we did not see any deletion less than 10 Mb, and the range of the deletion is between 11.6 to 103 Mb. It is probable that most deletions of a certain size (>3 Mb) can be detected with the current PGS array platforms. However, some small deletion syndromes, such as 17q21 deletion syndrome and 16p11.2 deletion syndrome, have deletions of about 500 kb [49], [50] and may not be detectable. Since some high resolution array platforms are matured for prenatal diagnosis, the undetection of microdeletions in PGS should be mainly due to the limitation of the primary whole genome amplification based on the biopsy materials. On the other hand, although the highest resolution of PGS array platform developed by NimbleGen may be able to detect deletions as small as 5 kb, such array platforms are still too expensive to be used for human PGS. By contrast, improved methods and higher resolution platforms may detect an increasing proportion of benign as well as pathogenic CNVs, thus many embryos may become non-transferrable. These conflicts may indicate that it is necessary to 1) use higher resolution array platform based on an improved WGA product that can detect at least 500 kb deletion, thus most (if not all) deletions in the preimplantation human embryos can be detected, or 2) follow up uneventfully until fetus births by ultrasound examination and prenatal diagnosis (if needed).

Based on the current information on the chromosomal deletion syndromes, it would appear that the occurrence of chromosome deletion syndrome is high in human population [15], [16], [38]. These deletion syndromes can occur on any chromosome, and we found that some deletion syndromes are detectable in the preimplantation embryos (Table 3). Most human aneuploidy embryos and euploid embryos with large size chromosomal deletion may not implant or reach live birth, but small chromosomal deletion in euploid embryos may not affect embryo implantation and/or live birth, thus some (perhaps most) may cause birth defects, such as delayed developmental and intellectual disability. As shown in Table 1, most chromosomal deletion syndromes may not have structural difference from normal healthy children, however, the common syndromes of these deletions are related to the delayed developmental and intellectual disability, thus these defects may not be easily noticed by physicians or parents.

Clinical genetic testing indicates that the incidence of developmental delay and/or intellectual disability in the general population is as high as 3% [51]. Although it is difficult to study the relationship between an embryo with deletion and genetic diseases in the child after live birth, the opportunity for a child to have the same deletion may be high if the embryo has. Based on our previous study, we found that chromosomal deletion in good quality human euploid embryos (blastocysts) is as high as 8%. If embryo transfer with good euploid blastocysts can establish 50% implantation and live birth rates, 4% of IVF babies may inherit the same chromosomal deletion syndrome, and this rate is almost equal to that by natural conception [51]. In the present study, we found that all 15 euploid blastocysts with various deletions had clinical significance if the embryos could cause live birth, and 5 out of 15 embryos had known genes in the deleted regions with developmental delay and intellectual disability. Although we did not find related genes with developmental delay and intellectual disability in other embryos, the deleted segment is quite large, and since many genes are involved, loss of these genes can cause other genetic diseases.

Chromosomal deletions can be caused by errors in chromosomal crossover during meiosis. The chromosomal deletion can also occur during translocation, chromosomal crossovers within a chromosomal inversion, and unequal crossing over and breaking without rejoining [1]. As shown in Table 1, some medium-sized deletions can lead to recognizable human disorders, e.g. Williams syndrome [52], while some just cause small problems and could not be noticed in early stage. For example, Y chromosome deletion that causes male infertility may be found only in the males when they become adult [53], [54].

In conclusion, our results indicate that the Agilent oligo array platform can be used for screening human embryos with the same sensitivity and accuracy as NimbleGen oligo array platform in terms of numerical and structural anomalies. The direct analysis of the data obtained with Agilent array platform can allow clinical physicians and scientists to further analyze the detailed chromosomal information, and such information is necessary to evaluate phenotypes of the related genes in the deleted region. Our results also indicate that all euploid human blastocysts with any chromosomal deletion are abnormal and should not be transferred to patients. Further development and combined application of advanced molecular techniques is necessary to detect small microdeletions (more than 500 kb) in human embryos produced by IVF.
